# Alpha-1 antitrypsin supplementation improves alveolar macrophages efferocytosis and phagocytosis following cigarette smoke exposure

**DOI:** 10.1371/journal.pone.0176073

**Published:** 2017-04-27

**Authors:** Karina A. Serban, Daniela N. Petrusca, Andrew Mikosz, Christophe Poirier, Angelia D. Lockett, Lauren Saint, Matthew J. Justice, Homer L. Twigg, Michael A. Campos, Irina Petrache

**Affiliations:** 1 Department of Medicine, Division of Pulmonary, Critical Care, and Sleep Medicine, National Jewish Health, Denver, Colorado, United States of America; 2 Pulmonary, Critical Care, Sleep and Occupational Medicine, Indiana University School of Medicine, Indianapolis, Indiana, United States of America; 3 Department of Medicine, Divisions of Hematology/Oncology, Indiana University School of Medicine, Indianapolis, Indiana, United States of America; 4 Department of Cellular and Integrative Physiology at Indiana University School of Medicine, Indianapolis, Indiana, United States of America; 5 Division of Pulmonary and Critical Care Medicine, University of Miami, Miami, Florida, United States of America; 6 University of Colorado, Anschutz Medical Campus, Aurora, Colorado, United States of America; University of Alabama-Birmingham, UNITED STATES

## Abstract

Cigarette smoking (CS), the main risk factor for COPD (chronic obstructive pulmonary disease) in developed countries, decreases alveolar macrophages (AM) clearance of both apoptotic cells and bacterial pathogens. This global deficit of AM engulfment may explain why active smokers have worse outcomes of COPD exacerbations, episodes characterized by airway infection and inflammation that carry high morbidity and healthcare cost. When administered as intravenous supplementation, the acute phase-reactant alpha-1 antitrypsin (A1AT) reduces the severity of COPD exacerbations in A1AT deficient (AATD) individuals and of bacterial pneumonia in murine models, but the effect of A1AT on AM scavenging functions has not been reported. Apoptotic cell clearance (efferocytosis) was measured in human AM isolated from patients with COPD, in primary rat AM or differentiated monocytes exposed to CS *ex vivo*, and in AM recovered from mice exposed to CS. A1AT (100 μg/mL, 16 h) significantly ameliorated efferocytosis (by ~50%) in AM of active smokers or AM exposed *ex vivo* to CS. A1AT significantly improved AM global engulfment, including phagocytosis, even when cells were simultaneously challenged with apoptotic and Fc-coated (bacteria-like) targets. The improved efferocytosis in A1AT-treated macrophages was associated with inhibition of tumor necrosis factor-α converting enzyme (TACE) activity, decreased mannose receptor shedding, and markedly increased abundance of efferocytosis receptors (mannose- and phosphatidyl serine receptors and the scavenger receptor B2) on AM plasma membrane. Directed airway A1AT treatment (via inhalation of a nebulized solution) restored *in situ* airway AM efferocytosis after CS exposure in mice. The amelioration of CS-exposed AM global engulfment may render A1AT as a potential therapy for COPD exacerbations.

## Introduction

Alveolar macrophages isolated from the lungs of cigarette smokers or those with CS-related COPD exhibit impaired clearance of pathogens (phagocytosis) and apoptotic cells (efferocytosis) [1, 2]. Whereas AM pathogen clearance is important for resolution of airway infection, removal of apoptotic cells ensures resolution of inflammation and repair [1]. The global dysfunction of AM engulfment in smokers and COPD patients may be most damaging during acute infectious exacerbations, episodes of increased airway infection and inflammation during which AM have to scavenge an increased load of both bacterial and apoptotic targets [3]. Given the high morbidity and mortality, as well as healthcare cost associated with COPD and COPD exacerbations [4, 5], therapies aimed at improving AM scavenging functions are of clinical importance.

AATD individuals have a higher risk of airways disease, including infectious exacerbations [6], suggesting an airway-protective effect of the anti-proteinase A1AT. In addition, in AATD patients augmentation of A1AT levels via intravenous administration of purified human protein reduces sputum levels of pro-inflammatory cytokines [7] and the severity of exacerbations [5, 8]. A protective airway effect of A1AT has also been demonstrated in non-AATD experimental model, where A1AT-overexpressing mice had reduced lung tissue damage following lung bacterial infections [9], and A1AT administration suppressed the AM release of pro-inflammatory mediators in CS-exposed mice [10]. The airway-protective effect of A1AT may indeed be due to its effects on AM and monocytes, since A1AT treatment profoundly affects peripheral blood monocyte derived macrophages (PBMDM) inflammatory response in a dose- and time-dependent manner. Whereas low A1AT concentrations and short exposure times increase TNF-α, IL-8, and IL-1β release, physiologic A1AT concentrations (corresponding to normal A1AT circulating levels; 500 μg/mL) or prolonged exposures (18 h) reduce pro-inflammatory cytokine secretion [11]. However, the effect of A1AT on AM engulfment of bacterial or apoptotic targets has not been reported.

Pathogens’ engulfment during phagocytosis begins with target recognition, a step that involves pathogen opsonization with complement or immunoglobulins and specific recognition by highly conserved complement receptors such as C1R, CD11b or the immunoglobulin receptor complex, such as FcγRI-III [12]. The abundance of these membrane receptors can be regulated by membrane proteases [13]. Since A1AT inhibits the sheddase TACE (ADAM17) [14], A1AT may play a specific role in maintaining the expression of FcγRI-III phagocytosis receptors on AM plasma membrane. This notion is supported by the finding that A1AT decreases the expression of several other phagocytosis receptors such as TLR4, and TLR2 on LPS-stimulated islet macrophages [14, 15].

Apoptotic targets’ engulfment (efferocytosis) occurs via a plethora of scavenger receptors that bind directly, or via a ligand (e.g. Gas-6 or protein S) to phosphatidylserine (PS) residues expressed on apoptotic cells’ plasma membrane [16]. The major scavenger receptors implicated in the AM engulfment of apoptotic targets include the mannose receptor (MAR, CD206), the macrophage receptor with collagenous structure (MARCO, SCARB-2), PS receptor (PSR), thrombospondin receptor (CD36), LDL receptor-related protein (CD91), hyaluronic acid receptor (CD44), and recognition molecules such as integrins [1]. These receptors may also be susceptible to protease cleavage that can in turn be modulated by A1AT, but these studies or the effect of A1AT on AM efferocytosis in general has, to our knowledge, not been reported yet.

Although AM from smokers or subjects with COPD are inefficient in clearing apoptotic cells, the engagement in efferocytosis polarizes them towards an anti-inflammatory phenotype, with increased local production of M-CSF, IL-4, IL-13, and IL-33 [17]. However, an excessive AM anti-inflammatory phenotype impairs phagocytosis and clearance of pathogens such as *S*. *pneumococcus* [18], decreasing antibacterial response in the murine lung. In turn, the effect of disproportionate AM phagocytic/ pro-inflammatory polarization on efferocytosis function has not been reported. During infectious exacerbations, the airways may simultaneously contain differently polarized AM populations whose function may be further confounded by active CS exposure. Furthermore, active CS exposure induces posttranslational modifications of the A1AT native molecule (e.g. cleavage and oxidation of the catalytic site), causing an “acquired” functional A1AT- deficient state [19–21]. These studies suggest that supplementation with native A1AT may be required to achieve functionally normal A1AT levels in COPD individuals, even if they do not have genetic AATD.

In this study we used complementary approaches to investigate whether exogenous supplementation of native A1AT enhances AM efferocytosis during CS exposure, without detrimental effects on phagocytosis. Our results indicate that A1AT treatment significantly improves AM efferocytosis and phagocytosis, associated with TACE-inhibition and increase in specific scavenging receptor membrane expression. Moreover we demonstrate that when concomitantly exposed to Fc-coated “bacteria-like” and apoptotic targets, A1AT-treated AM exhibit increased overall scavenging activity.

Preliminary results of our studies have been previously presented in the form of abstracts.

## Material and methods

### Reagents

Reagents, including LPS and rabbit anti-JMJD6 (PSR) antibody, were purchased from Sigma-Aldrich (St. Louis, MO, USA) unless otherwise specified. Aralast NP and Prolastin C was from Baxter International Inc. (Chicago, IL, USA) and Grifols (LA, CA, USA), respectively. Phorbol 12-myristate 13-acetate (PMA), rabbit anti-MAR antibody, rabbit anti-iNOS antibody, rabbit polyclonal anti-GAPDH, mouse monoclonal anti-vinculin were from Abcam (Cambridge, MA, USA), rabbit anti-SRB-2 antibody was from Santa Cruz Biotechnology (Dallas, TX, USA), mouse anti-human CD3 was from BD Biosciences (Franklin Lakes, NJ, USA), recombinant rat IL-4 was from ProSpec-Tany TechnoGene (Ness-Ziona, Israel), and TNF-α Protease Inhibitor-1 (TAPI-_1_) was from ESD Millipore (Billerica, MA, USA).

### Human subjects

Healthy non-smokers (n = 4) and smokers (n = 6) subjects were consented for research bronchoscopy. Written consent was obtained per study protocol. IRB at Indiana University, Indianapolis, IN approved this protocol. COPD was defined based on GOLD 2007 criteria using the FEV1/FVC ratio <70% to define obstruction and post-bronchodilator FEV1 to define the obstruction severity (mild to moderate <80% but >50%; severe to very severe <50% predicted FEV1). Individuals classified as GOLD stage 0 were active smokers with FEV1/FVC>70% but with clinical symptoms of chronic bronchitis or dyspnea on exertion. Current smoker (n = 2), ex-smoker (n = 1), and healthy non-smoker (n = 1) de-identified human lungs that were not used for organ-transplantation with a PaO_2_/FiO_2_ ratio of >225, a clinical history and chest X-ray diagnosis that did not indicate infection, and limited time on a ventilator from the University of Colorado Donor Alliance were used for ex-vivo bronchoalveolar lavage (BAL). We noted the age, gender, smoking history, and cause of death. The Committee for the Protection of Human Subjects at National Jewish Health, Denver, CO approved this research. AATD individuals (PiZZ genotype, n = 3) were enrolled in the clinical study “Effect of Double Dose of Alpha 1-antitrypsin Augmentation Therapy on Lung Inflammation” at the University of Miami, Miami, FL (ClinicalTrials.gov Identifier: NCT01669421). AATD patients underwent bronchoscopy with BAL after a single- (60 mg/kg, weekly administration at visit 1 and 3) or double dose (120 mg/kg, weekly administration at visit 2) A1AT therapy (Zemaira, CSL Behring). Written consent was obtained per study protocol. IRB at the University of Miami, Miami, FL approved this research.

During research bronchoscopy bronchoalveolar lavage (BAL) of right middle lobe or lingua was performed with 100–240 mL sterile 0.9% saline. The BAL cells were pelleted by centrifugation and then plated in RPMI media supplemented with 1% non-essential amino acids, 2% sodium pyruvate, 20mM Hepes, penicillin (100 U/ ml), and streptomycin (0.1 mg/ml) for 2 h before being used in experiments. The non-adherent cells were discarded. The adherent human AM were used in assays as described below.

### Animal experiments

All experiments were conducted in compliance with the Institutional Animal Care and Use Committee guidelines of Indiana University. Male Sprague-Dawley rats (8-16-week old, 400–600 g, n = 60) were obtained from Charles River Laboratories (Wilmington, MA, USA). BAL was performed as previously described. Briefly, rats were anesthetized with isoflurane and euthanized by bilateral pneumothorax. After neck dissection and midline tracheostomy the BAL needle was secured in place and 10 mL cold sterile 0.9% NaCl was instilled and recovered three times. Cells in the BAL fluid were pelleted by centrifugation (300xg, 8 min) and then maintained in DMEM supplemented with 10% heat-inactivated FBS, 1% penicillin and streptomycin. Prior to experiments and treatments the medium was changed to low-serum (2% FBS) DMEM.

Eight to ten-week-old male C57B/l6 mice from Charles River Laboratories (Wilmington, MA, USA) were exposed to cigarette smoke in a total body exposure chamber (TE-10Z, Teague Enterprises, Woodland, CA, USA) for 3 hours (n = 13) or for 5 hours/day, 5 days/week, for 6 months (n = 10) or to similar duration of ambient air (n = 21). Research-grade cigarettes (1R3F, Kentucky Tobacco Research and Development Center, Lexington, KY, USA) were smoked at a rate that achieved 90–120 ng/m^3^ microparticles in the exposure chamber. The mice received aerosolized PBS or human A1AT using a micropump nebulizer (AeronebLab, Aerogen, Inc.) 24 h prior to a brief (3 h) episode of cigarette smoke exposure or daily for the last 7 days of a long-term (6-months) CS exposure. The day of experiment the mice received intratracheal apoptotic thymocytes via oropharyngeal aspiration (10X10^6^ cells/mouse). After 1 h mice were anesthetized with isoflurane, euthanized by bilateral pneumothorax, and BAL was performed.

### Cells

Primary human AM obtained from the BAL fluid of healthy subjects or smokers were cultured in RPMI-1640 supplemented with 1% non-essential amino-acids, 2% sodium pyruvate, 20mM Hepes, and penicillin (100 U/ ml).

Primary human AM obtained from the BAL fluid of AATD individuals underwent centrifugation (300 g, 8 min), were washed two times with sterile PBS, and lysed using Triton-X lysis buffer. Cell lysates were saved at -80°C until used for Western blotting.

Primary rat AM obtained from BAL of Sprague-Dawley rats were cultured in DMEM medium (Life Technologies, Grand Island, NY, USA) containing 10% heat-inactivated fetal bovine serum (FBS), and penicillin (100U/mL) and streptomycin (0.1 mg/mL) and acclimatized for 2–3 days in the incubator at 37°C, 5% CO_2_. One hour prior to the A1AT treatments the primary rat cells were changed to low serum (2% FBS) medium.

Rat AM (cell line NR8383, ATCC Manassas, VA) were cultured in Ham's F12K medium (ATCC) containing L-glutamine (2 mM), sodium bicarbonate (1.5 g/L), and 15% heat-inactivated FBS and maintained in the incubator at 37°C, 5% CO_2_. One-day prior the experiments the NR8383 cells were plated in 6-well plates (0.5 X 10^6^ cells/well) or 100 mm Petri dishes (2X10^6^ cells/dish). One hour prior to the A1AT treatments the NR8383 AM were changed to low serum (2% FBS) medium.

Human T-cells (acute T-cell leukemia cell line, Jurkat, ATCC) were cultured in RPMI-1640 supplemented with 10% heat-inactivated FBS, penicillin (100 U/ ml), and streptomycin (0.1 mg/ml).

Human monocytes (cell line THP-1, ATCC) were cultured in RPMI-1640 supplemented with 10% heat-inactivated FBS, 2 mercaptoethanol (0.05 mM), penicillin (100 U/ ml), and streptomycin (0.1 mg/ml). Monocyte-derived macrophages were obtained by differentiation with PMA (100nM, 72 h).

### CS extract preparation

Aqueous CS extract (100%) was prepared from filtered research-grade cigarettes (1R3F, Kentucky Tabaco Research and Development Center, Lexington, KY, USA). Similarly, the air control extract (AC) was prepared by bubbling ambient air into sterile PBS, as previously described [22]. The final concentrations of CS and AC extracts used in cell culture experiments ranged from 3% (primary rat AM) to 10% (NR8383 and THP-1 cell lines). At these concentrations, CS extract inhibited AM proliferation, but did not affect AM viability, as assessed by MTT (3-(4,5-dimethylthiazol-2-yl)-2,5-diphenyltetrazolium bromide) proliferation assay or Annexin V staining (data not shown). Exposure time to AC or CS extract was 4 h. Post-exposure the cell culture media was removed and low serum media (2% FBS) with or without A1AT (100 μg/mL) was added.

### Polymerized A1AT preparation

Polymers of A1AT were prepared as previously described by incubating Aralast NP A1AT protein (5 mg/mL) in phosphate buffered saline (PBS) at 60°C for 2 h [21, 23].

### Efferocytosis assays

*In situ* efferocytosis was assessed in mice that received via oropharyngeal aspiration fluorescently labeled apoptotic thymocytes (10X10^6^ cells/mouse) [24]. The efferocytosis index was measured after 1 h using flow cytometry, and was expressed as the percentage of AM recovered in the BAL fluid that engulfed labeled thymocytes. *Ex vivo* efferocytosis assay was performed as previously described [25]. Briefly, AM were co-incubated with apoptotic targets (1:5, unless otherwise specified) for 1 h at 37°C. Apoptotic targets were Jurkat cells (T-cell line) that were fluorescently-labeled with Cell Tracker Orange (CTO, 0.5 mM, 30 min, 37°C, Life Technologies, Grand Island, NY, USA), and then exposed to UV radiation (30,000 μJ/cm^2^) using a HL-2000 HybriLinker, followed by incubation for 3.5 h at 37°C, 5% CO^2^ in serum-free media. At the end of co-incubation (1 h) with apoptotic Jurkat cells, AMs were collected in flow-cytometry tubes. The extracellular fluorescence (of membrane-bound but non-engulfed apoptotic targets) was quenched with trypan blue (Sigma; 1000μl; 0.04% in PBS)[25, 26]. Cells were then fixed in 1% paraformaldehyde. Engulfment efficiency was measured by flow cytometry using Cytomics FC500 cytofluorimeter with CXP software (Beckman Coulter, Fullerton, CA).

### Phagocytosis assay

Phagocytosis assay was performed by co-incubation of AM with phagocytic “Fc-coated” opsonized targets for 1 h at 37°C at 1:5 ratio, unless otherwise specified. We used two types of phagocytic targets: a) fluorescently-labeled latex beads (Sigma, Saint Louis, MO, USA) coated with 1% BSA for 1 h at 4°C and then incubated with rabbit anti-bovine albumin antibody (1:500, 30 min, 37°C; or b) Cell Tracker Green (CTG, 0.5mM, 30 min, 3°C, Life Technologies, Grand Island, NY, USA)-labeled Jurkats coated with mouse IgG2a anti-human CD3 antibody (10 μl/mL, 30 min, 37°C). At the end of co-incubation with phagocytic targets, AM were collected in flow-cytometry tubes and engulfment was measured by flow cytometry.

### TNF- α converting enzyme (TACE) activity

TNF- α converting enzyme (TACE) activity was measured in the human monocyte THP-1 membrane fractions using SensoLyte 520 TACE Activity Assay Kit (Anaspec, Fremont, CA, USA) as per manufacturer’s instructions.

### Western blotting

Cells were harvested in RIPA lysis buffer. Cytosolic and membrane fractions were extracted with the membrane protein extraction kit (Biovision, Milpitas, CA, USA), using the manufacturer protocol. Whole cell lysates or membrane fractions were loaded in equal amounts, as determined by BCA protein analysis (Pierce). We used equal volumes of cell supernatants after concentration using 50μm columns (Millipore, Tullagreen, Carrigtwohill, Co. Cork, IRL). Proteins were separated by SDS-PAGE and transferred onto a PVDF membrane followed by immunoblotting. The chemiluminescent signal was detected using ECL-plus (Amersham, NJ, USA) and normalized using anti-vinculin (1:5000) or -GAPDH (1:5000) antibody.

### ELISA

Supernatants from AM cultures were concentrated using VWR columns (VWR, Radnor, PA, USA). TNF-α was measured by ELISA (R&D Systems, Minneapolis, MN, USA), as per manufacturer’s protocol.

### Statistical analysis

Statistical analysis was performed in Prism (GraphPad Software, San Diego, CA), using unpaired Student t-test, 1-way or 2-way ANOVA, as appropriate. Statistical significance was accepted at p<0.05.

## Results

### Effect of native and polymerized A1AT on efferocytosis in CS-exposed AM

**Fig 1:**
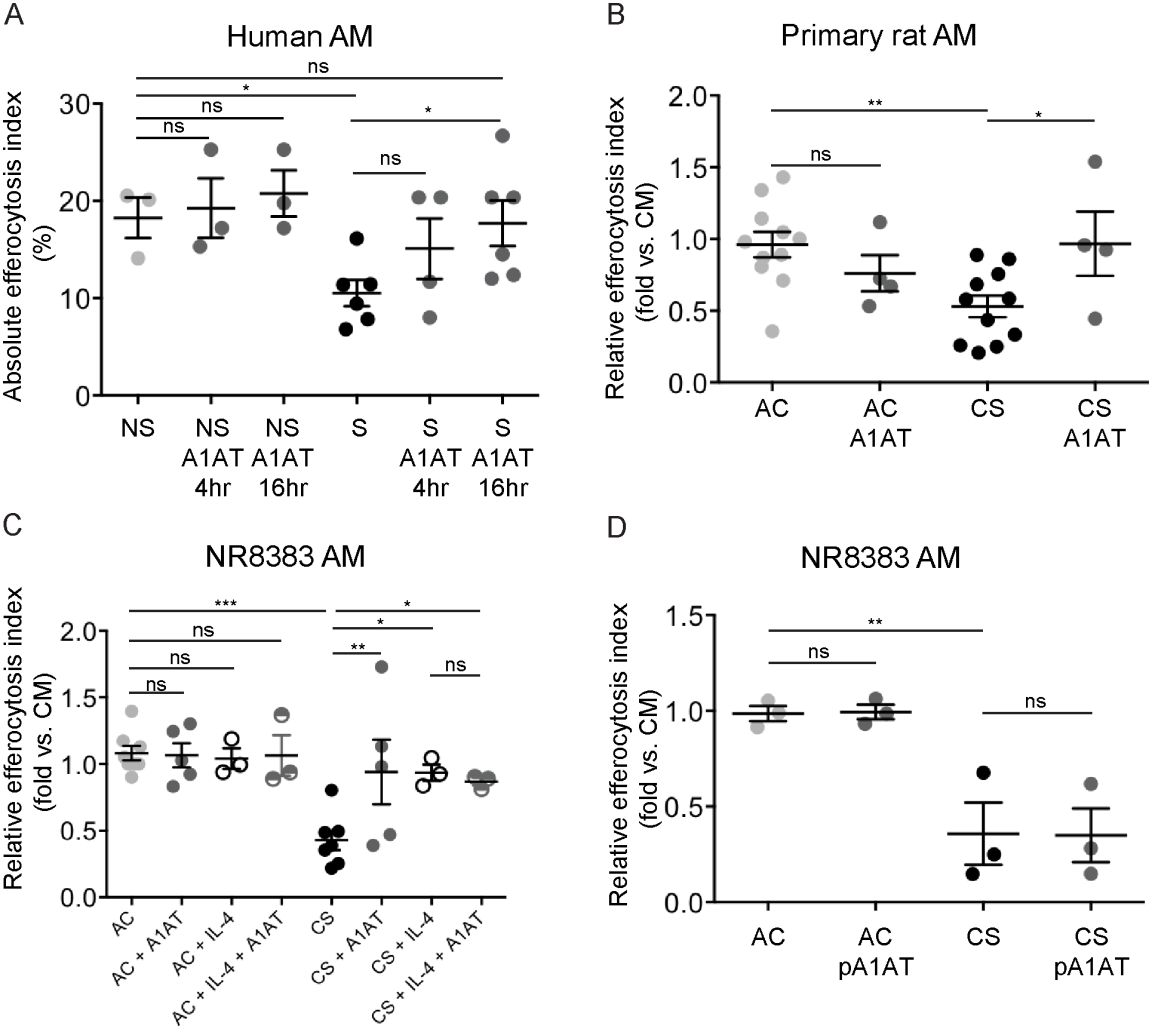
Native and polymerized A1AT *ex-vivo* effect on CS-exposed AM efferocytosis. **A**. Absolute efferocytosis index representing % of cells that engulfed fluorescently labeled apoptotic targets among AM isolated from active smokers (dark circles) compared to those from healthy non-smokers (light gray circles). Native A1AT (Aralast NP, 100 μg/mL, 16 h) significantly increased efferocytosis in AM from smokers (dark gray circles), but not in those from healthy non-smokers (gray squares). 1-way ANOVA, Sidak’s multiple comparisons test, * p<0.05. **B-C**. Relative efferocytosis index representing % of AM that engulfed fluorescently labeled apoptotic targets following indicated exposures, when compared to those exposed to control media. Primary rat AM (**B**) or NR8383 AM (**C**) were exposed *ex-vivo* to AC or CS (4h, 3% or 10%, respectively, 4 h), native A1AT (Aralast NP, 100 μg/mL, 16 h), and compared to IL-4 (20 ng/mL, 72 h). **D**. Polymerized A1AT (Aralast NP, 100 μg/mL, 16h) has no effect on CS-exposed NR8383 AM efferocytosis. Data are presented as mean ± SEM, 1-way ANOVA, Sidak’s multiple comparisons test, * p<0.05, ** p<0.001, *** p<0.0001.

Primary AM isolated via BAL from individuals who were either never smokers or active smokers (demographic and clinical characteristics listed in Table 1) were tested for efferocytosis by co-incubation with apoptotic targets. As expected, AM from active smokers exhibited significantly impaired efferocytosis compared to AM from non-smokers (Fig 1A). The effect of A1AT on efferocytosis was tested after supplementation of AM cultures with purified human A1AT (100 μg/mL, 4 h or 16 h) prior to the addition of apoptotic cells. A1AT treatment for 16 h significantly increased efferocytosis of AM isolated from active smokers by ~50% (Fig 1A). Of note, shorter A1AT treatment (4 h) was insufficient to improve efferocytosis of these cells (Fig 1A). Treatment with A1AT for either 4 h or 16 h had no additional effect on efferocytosis efficiency of AM isolated from non-smokers, suggesting that either efferocytosis was maximal in cells from healthy individuals, or that cells from smokers were more likely to respond to A1AT treatment.

**Table 1:** Patient demographic, clinical, and functional characteristics.

	Healthy	Smokers at risk	COPD	AATD (PiZZ)
Number	5	6	3	3
Gender (M, %)	80	50	100	100
Age (years)	40 ± 11.3	37 ± 12.1	52 ± 3.7	53.5 ± 10.5
Race (W, %)	40	83	33	100
Smoking status (ex-smoker/never smoker)	0/2	6/0	2/1	2/1
Smoking history (PY)	1.5 ± 2	12.5 ± 4.5	29.3 ± 7	30 ± 21
FEV_1_ (%predicted)	NA	NA	73 ± 10.2	48.1 ± 14.2
GOLD stage (0/1-2/3-4)	NA	6/0/0	0/3/0	0/1/2
A1AT (mg/dL) Visit 1 Visit 2 Visit 3	NA	NA	NA	73.0 ± 5.0 126 ± 12.7 71.0 ± 6.1
BAL fluid cell count (X10^7^ cells/mL)	2.6 ± 1.1	15.1 ± 10.3	NA	NA

Data are presented as mean ± SD or percentage (%). Abbreviations: AATD: alpha-1 antitrypsin deficiency; PiZZ—homozygous for A1AT gene Z mutation; PY: pack-year; FEV_1_: forced expiratory volume in 1 s; GOLD: the Global initiative for chronic Obstructive Lung Disease; A1AT: alpha-1 antitrypsin; BAL: broncho-alveolar lavage

This effect of A1AT on AM efferocytosis was recapitulated in models of *ex vivo* CS- exposure. As we previously reported, CS (3%-10% v/v, 4 h) significantly inhibited efferocytosis in primary rat AM (Fig 1B) and in the NR8383 AM (Fig 1C). Immediately after CS-exposure A1AT treatment (100 μg/mL, 16 h) significantly improved efferocytosis of primary (Fig 1B) and NR8383 (Fig 1C) AM exposed to CS.

To compare the A1AT effect on CS-exposed AM efferocytosis with a known efferocytosis activator, we used IL-4, a potent activator of AM polarization towards an anti-inflammatory phenotype. Indeed, IL-4 treatment restored the efferocytosis function of CS-exposed AMs to that of control cells (Fig 1C). A1AT treatment (100 μg/mL, 16 h) increased AM efferocytosis index by a similar level of magnitude (Fig 1C), but had no additive effect on IL-4 stimulated, CS-exposed AMs (Fig 1C).

We then compared the effect of polymerized A1AT (pA1AT) with that of native A1AT on CS-exposed AM efferocytosis. A1AT polymers were generated by heating A1AT (5mg/mL) in 10 mL sterile PBS at 60°C for 2 h [21, 23]. In contrast to the effect of native A1AT, similar concentrations and duration of exposure to pA1AT failed to rescue CS-exposed NR8383 AM efferocytosis (Fig 1D).

### Effect of nebulized A1AT on AM *in situ* efferocytosis in mice exposed to CS

**Fig 2:**
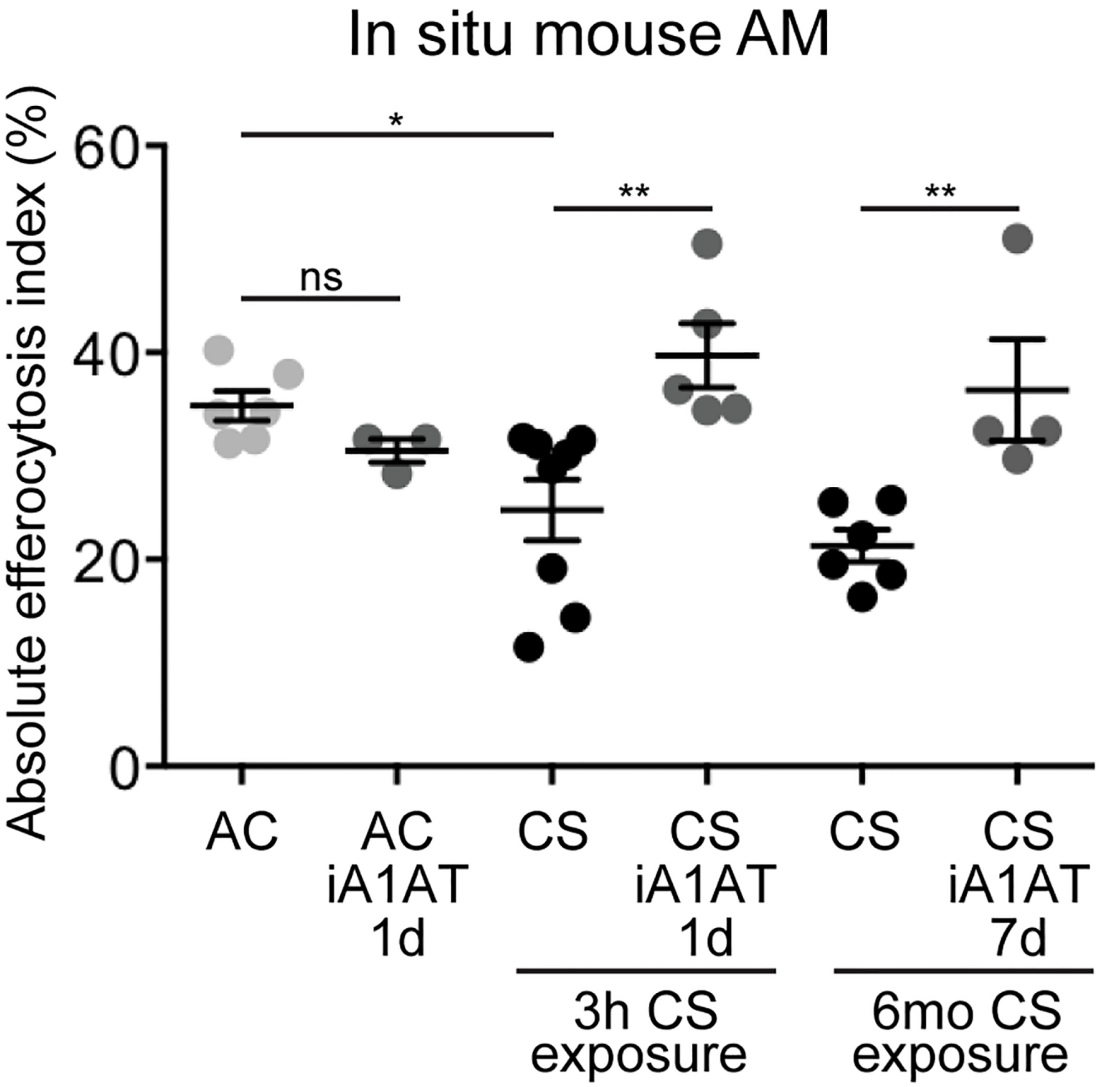
Nebulized human A1AT effect on *in situ* efferocytosis in CS-exposed mice. Nebulized PBS or A1AT (Aralast NP, 1mg/kg body weight, one individual dose or daily dose for 7 days, respectively) were administered to C57Bl/6 mice prior to acute (3 h) or during the final week of chronic (6 mo) CS-exposure. In situ efferocytosis was quantified in the BAL fluid after orotracheal instillation and co-incubation (1 h) with fluorescently labeled apoptotic thymocytes. Data are presented as mean ± SEM, 2-way ANOVA, * p<0.05, ** p<0.001.

To investigate the effect of A1AT on AM efferocytosis *in vivo*, C57BL/6J mice were exposed to CS for short- (3 h) or long term (6 months), followed by challenge with exogenous apoptotic targets. As previously reported [24], CS exposures decreased AM efferocytosis compared with ambient air-exposed mice (Fig 2). Native A1AT alveolar delivery was achieved via inhalation of a nebulized solution [27]. A1AT (25 mg/mouse; ~0.1 mg/kg) either as a single administration 24 h prior to brief CS-exposure, or as a daily administration for seven consecutive days during last week of a long-term CS-exposure significantly increased AM engulfment compared to mice that received control vehicle (PBS) treatments (Fig 2). These results validated our *ex-vivo* results of improved efferocytosis in CS- and A1AT-treated AM. Since efferocytosis requires the participation of specific recognition receptors, and since recent reports implicate A1AT in the expression of several plasma membrane receptors [14, 28], we next investigated whether A1AT alters the expression of efferocytosis receptors.

### Effect of A1AT on AM efferocytosis receptors expression

**Fig 3:**
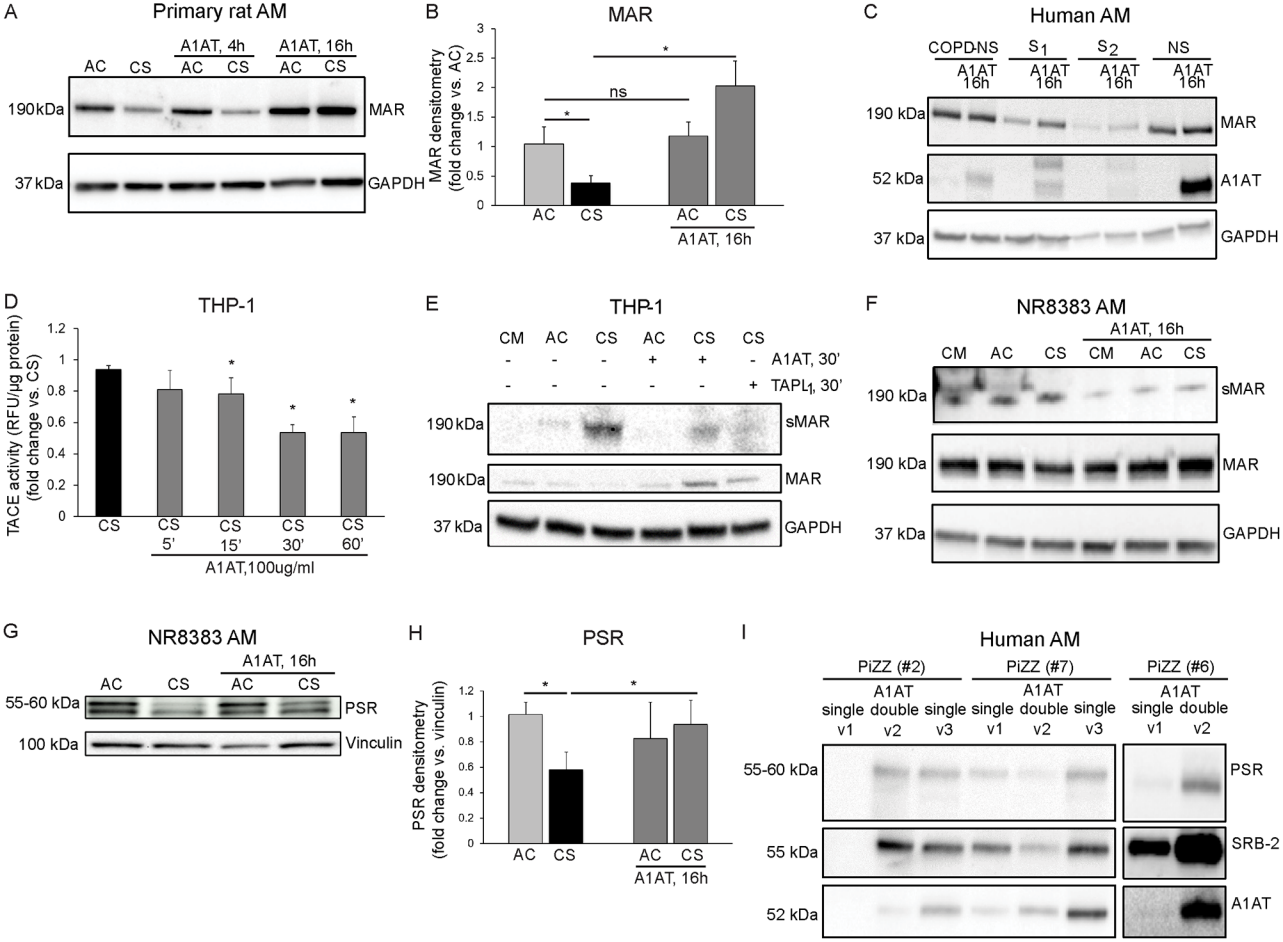
Native A1AT effect on efferocytosis receptors expression and ADAM-17 (TACE) activity in CS-exposed AM. **A-B.** Representative immunoblot (**A**) and densitometry (**B**, n = 4) of MAR expression in CS-exposed (3%, 4 h) and A1AT-treated (Aralast NP, 100 μg/mL, 16 h) primary rat AM. 1-way ANOVA, Sidak’s multiple comparisons test, * p<0.05. **C.** Representative immunoblots (n = 4) of MAR expression in human AM isolated from the BAL of non-smoker and smoker individuals prior and after *ex-vivo* A1AT treatment (Prolastin C, 100 μg/mL, 16 h). **D.** A1AT treatment (Prolastin C, 100 μg/mL) time-dependently decreases TACE activity in the CS-exposed (10%, 4 h) THP-1 membrane fraction. **E.** Representative immunoblot of MAR expression in the CS-exposed THP-1 cell lysates and supernatants after treatment with A1AT (Prolastin C, 100 μg/mL, 30 min) or with a pharmacological inhibitor of TACE (TAPI-1, 50 μM, 30 min). **F**. Representative immunoblot of MAR expression in the CS-exposed NR8383 AM cell lysates and supernatants after A1AT treatment (Prolastin C, 100 μg/mL, 16 h). The results are representative of 3 independent experiments. **G-H.** Representative immunoblot (**G**) and densitometry (**H**, n = 4) of PSR expression in CS-exposed (10%, 4 h) and A1AT-treated (Aralast NP, 100 μg/mL, 16 h) NR8383 AM membrane fractions. **I.** Representative immunoblots (n = 3) of PSR and SRB-2 expression in PiZZ-AM (13 μg protein equally loaded in each lane) after A1AT augmentation therapy (Zemaira, CSL Behring, 60 mg/kg single dose or 120 mg/kg double dose). Note that doubling the weekly A1AT dose (visit 2) increases PiZZ-AM A1AT intracellular abundance and PSR and SRB-2 expression levels vs. A1AT single dose (visit 1), effect that persist as carried over effect after resuming single A1AT dose (visit 3).

A1AT modulation of neutrophil chemotaxis has been reported to occur via controlling membrane expression of the Fc receptor FcγRIIIb [14], however A1AT effect and the mechanism involved in membrane expression of efferocytosis receptors are not known. We first measured the A1AT effect on MAR membrane expression in primary rat AM during *ex vivo* CS-exposure. Under baseline conditions, A1AT treatment did not show a significant effect on MAR expression on rat AM plasma membrane (data not shown). However, after CS exposure primary rat AM exhibited decreased MAR plasma membrane expression (Fig 3A and 3B) that was significantly increased after A1AT (100 μg/mL, 16 h) treatment (Fig 3A and 3B). The A1AT effect on MAR membrane expression was recapitulated in human AM isolated from the BAL fluid of explanted lungs of non-smokers and active smokers individuals. AM recovered from active smokers, but not AM recovered from non-smoker or ex-smokers with COPD individuals, showed increased MAR expression after *ex vivo* A1AT (100 μg/mL, 16 h) treatment (Fig 3C).

An important mechanism to retain membrane receptors at plasma membrane is inhibition of membrane sheddase TACE (ADAM-17) [29, 30]. We and others have shown that A1AT inhibits TACE activity at plasma membrane in structural, e.g. endothelial cells [28] and immune cells, e.g. neutrophils [14]. A1AT treatment inhibited in a time dependent manner TACE activity in the membrane fraction of CS-exposed (10%, 4 h) THP1-derived macrophages (Fig 3D). Accordingly, treatment with A1AT (100 μg/mL, 30 min) or TNF-α Protease Inhibitor-1 (TAPI-_1_, 50 μM/mL, 30 min) significantly decreased soluble MAR (sMAR) shedding in the supernatant of CS-exposed THP-1-derived macrophages while increasing MAR membrane expression (Fig 3E). These results were recapitulated in CS-exposed (10%, 4 h) NR8383 AM treated with A1AT (100 μg/mL, 16 h) (Fig 3F).

Since several other specific (e.g. PSR, CD91) and facultative (e.g. SCARB-2, CD36, or CD44) efferocytosis receptors are down regulated following *in vivo* and *ex vivo* CS-exposure [31, 32], we next investigated whether A1AT supplementation increases their expression in these conditions. We examined AM membrane expression of PSR and SCARB-2 after *ex vivo* or *in vivo* A1AT therapy in CS-exposed NR8383 AM and in AATD patients. After CS exposure NR8383 AM exhibited decreased PSR plasma membrane expression (Fig 3G and 3H) that was significantly increased after A1AT (100 μg/mL, 16 h) treatment (Fig 3G and 3H). We noticed a similar *in vivo* A1AT effect on PiZZ AM PSR and SRB-2 membrane expression where A1AT double dose therapy increased efferocytosis receptors (visit 2) compared to levels after single dose A1AT therapy (visit 1). The A1AT double dose effect persisted as carried over effect after resuming single A1AT dose (visit 3) (Fig 3I).

### Time-dependent effects of A1AT on phagocytosis and global scavenging function of AM concomitantly exposed to Fc-coated and apoptotic targets

Since A1AT improved both AM efferocytosis and the expression of efferocytosis receptors during CS exposures, A1AT treatment may impart AM an anti-inflammatory functional phenotype. Previous studies have reported that AM anti-inflammatory programs activated by efferocytosis have the potential to undesirably inhibit bacteria phagocytosis and killing [18]. We therefore interrogated the effect of A1AT on AM engulfment of Fc-coated targets, on inducible NO synthase (iNOS) expression and TNF-**α** secretion, traits associated with AM’s ability to effectively phagocytize bacterial targets.

**Fig 4:**
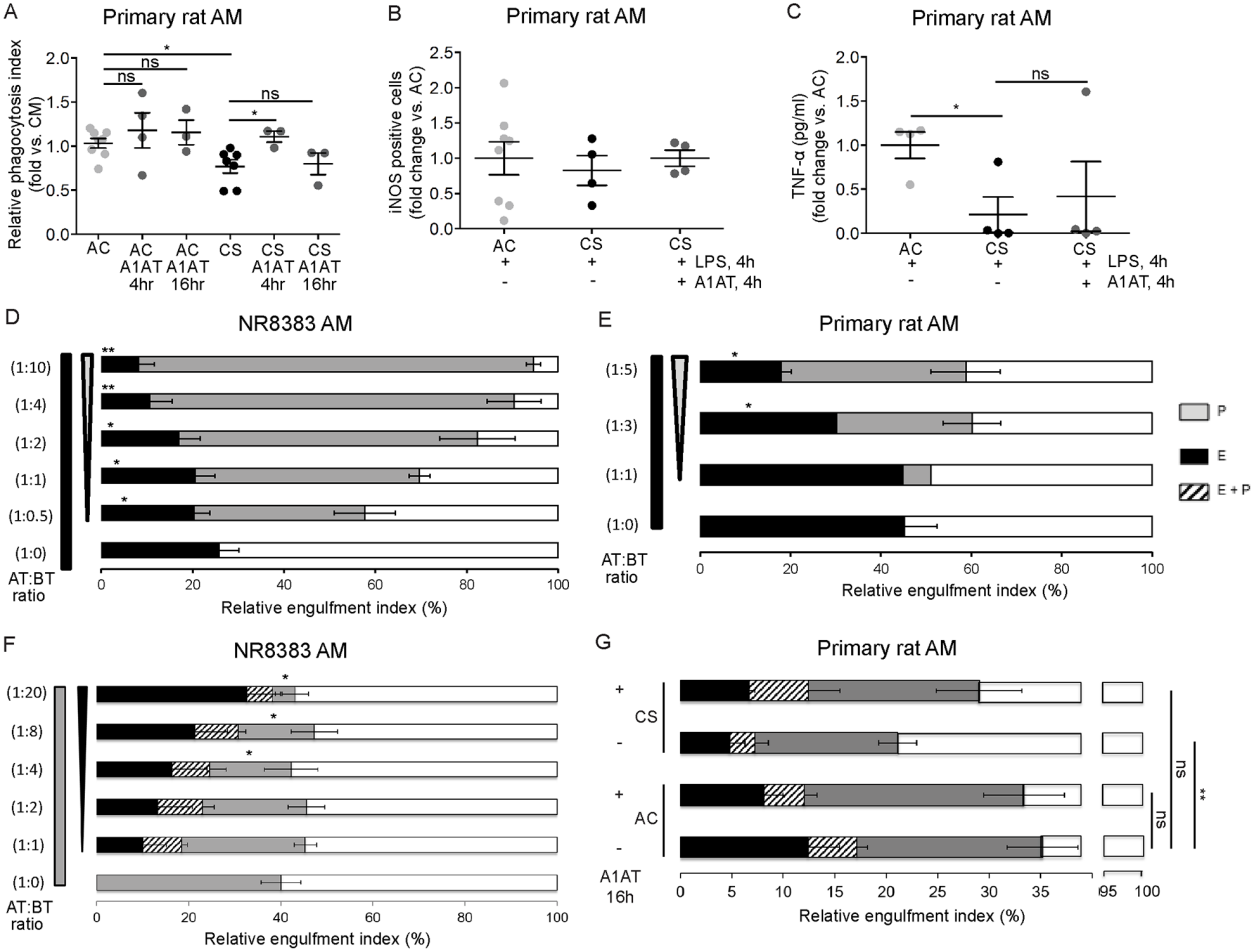
Native A1AT effect on CS-exposed AM phagocytosis and global scavenging function following concomitant Fc-coated and apoptotic targets exposure. **A-B.** Relative phagocytosis index representing % of primary rat AM that engulfed fluorescently labeled Fc-coated targets after CS-exposure (10%, 4 h) and A1AT treatment (Aralast NP, 100 μg/mL, 4 h), compared to AM engulfment in control media. 1-way ANOVA, Sidak’s multiple comparisons test, * p<0.05. Note the lack of significant effect on phagocytosis at baseline (AC) or during CS-exposure following 16 h of A1AT treatment. **B-C**. iNOS expression (**B**) and TNF-α secretion (**C**) in primary rat AM exposed to CS (3%, 4 h) and treated with LPS (100 ng/mL, 4 h) and A1AT (Aralast NP, 100 μg/mL, 4 h). **D.** Higher Fc-coated to apoptotic targets ratio (0:1 to 10:1) dose-dependently inhibits NR8383 AM efferocytosis. **E.** Higher mouse anti-human CD3^+^ CTG-labeled Jurkat T-cells (Fc-coated targets) to apoptotic targets ratio (0:1 to 5:1) dose-dependently inhibits primary rat AM efferocytosis. **F.** Higher apoptotic to Fc-coated targets ratio (0:1 to 24:1) dose-dependently inhibits NR8383 AM phagocytosis. **G.** Overall engulfment [efferocytosis (E), phagocytosis (P), and efferocytosis + phagocytosis (E+P)] of CS-exposed and A1AT-treated (Aralast NP, 100 μg/mL, 16 h) primary rat AM when concomitantly exposed to apoptotic and Fc-coated targets at 1:1 ratio. 1-way ANOVA, Sidak’s multiple comparisons test, * p<0.05. Data are presented as mean ± SEM.

A1AT treatment significantly improved phagocytosis of CS-exposed AM, as measured by percentage engulfed fluorescently labeled Fc-coated, bacteria-like targets following 4h of A1AT supplementation (Fig 4A). However, this beneficial effect did not persist at 16 h following A1AT treatment (Fig 4A). Despite significant improvement in AM phagocytosis function, A1AT treatment did not increase pro-inflammatory intracellular iNOS expression and TNF-α secretion of LPS- and CS-exposed primary rat AM (Fig 4B and 4C).

We next determined the effect of A1AT on AM scavenging function in a complex environment, where both apoptotic and Fc-coated targets are present. While an inhibitory effect of efferocytosis on phagocytosis has been previously described [18], the effect of Fc-coated target phagocytosis on efferocytosis has not been reported. We measured the reciprocal effect of Fc receptor-mediated engulfment (phagocytosis) on AM efferocytosis and determined the baseline AM global scavenging function, defined as the proportion of AM that performed any target engulfment. In NR8383 AM, phagocytosis of Fc-coated targets suppressed in a dose-dependent manner the proportion of AM that performed exclusively efferocytosis of apoptotic targets (Fig 4D). To test whether the phagocytosis of larger Fc-coated targets has similar effects, primary rat AM were concomitantly incubated with CTG-labeled Jurkat T-cells coated with IgG2a anti-human CD3 (larger size Fc-coated targets) and apoptotic targets. We noticed the same marked inhibitory effect of phagocytosis on efferocytosis (Fig 4E). When AM were treated with increasing apoptotic targets, as reported, efferocytosis dose-dependently inhibited Fc receptor-mediated AM engulfment (phagocytosis) of Fc-coated targets (Fig 4F).

E*x-vivo* CS exposure of primary rat AM simultaneously challenged with Fc-coated and apoptotic targets decreased phagocytosis, efferocytosis, and the global engulfment of these cells (Fig 4G). Treatment with A1AT (100 μg/mL, 16 h) treatment significantly increased the global scavenging function of CS-exposed AM concomitantly challenged with Fc-coated and apoptotic targets (Fig 4G).

## Discussion

Our study identified that treatment with A1AT significantly improves efferocytosis, phagocytosis, and overall scavenging function of CS-exposed dysfunctional AM. This finding may have clinical relevance to smokers and individuals with COPD with impaired AM scavenging function, which perpetuates pathogen persistence and lower airways bacterial colonization linked to chronic inflammation, tissue damage, and frequent exacerbations [3]. These results indicate that native A1AT protein can be considered along with peroxisome proliferator-activated receptor γ agonists, macrolide antibiotics, corticosteroids, statins, and anti-oxidants as enhancer of AM engulfment of either pathogens or apoptotic targets during CS exposure [1]. Whereas exogenous A1AT did not further augment the engulfment function of healthy AM, a role of endogenous native A1AT in acute inflammatory and repair phases of airway infections in otherwise healthy individuals remains to be investigated. However, in smokers or in AATD, where the function and levels, respectively, of endogenous A1AT may be suboptimal to stimulate airway bacteria and apoptotic cell clearance, A1AT supplementation may be required to achieve functional A1AT levels that restore AM engulfment.

A1AT stimulated efferocytosis of CS-exposed AM not only in *ex vivo* models, but also *in vivo*, following exogenously administered apoptotic targets in CS-exposed C57BL/6J mice. Since both increased efferocytosis and inhibition of apoptosis lower the abundance of apoptotic cells in tissues, our current results are congruent with our previous reports of decreased apoptotic cells in lungs of animals exposed to pro-apoptotic stimuli and treated with exogenous A1AT [33].

To our knowledge, we revealed for the first time that A1AT’s stimulatory effect on efferocytosis was associated with increased abundance of AM receptors that recognize apoptotic targets, recognition required as first step for successful completion of efferocytosis. Unlike complement- or Fc-mediated phagocytosis, which involve highly conserved, receptor-specific engulfment of pathogens [2, 34], efferocytosis of apoptotic targets involves a wide array of receptors—MAR, SRB-2, PSR, CD36, LDL-rp, CD44 and bridging molecules—Milk-fat-globule-factor (MFG-E8), Gas6, thrombospondin, β2 glycoprotein-I, protein S, and annexin I [1]. Consistent with studies by Hodge *et al*., we found decreased levels of MAR, PSR, and SRB-2 on AM exposed to CS, associated with impaired AM efferocytosis in active smokers and COPD individuals [31, 35, 36]. A1AT treatment significantly increased these receptor levels in human AM from active smokers, AATD individuals, and in CS-exposed primary rat and NR8383 AM. Moreover, A1AT treatment decreased sMAR shedding from the plasma membrane of CS-exposed AM, consistent with TACE-inhibition by the serpin, a non-canonical function shown by others and us in PMN [14], endothelial cells [28], respectively, and now demonstrated here in AM. While this association does not prove causality, the evidence that MAR is cleaved by TACE [30], that TACE is inhibited by A1AT [14, 28], and that A1AT decreases sMAR shedding and increases MAR’s plasma membrane abundance highly suggests that TACE inhibition by A1AT is a central mechanism accounting for enhanced efferocytosis. However, the mechanisms by which A1AT enhances Fc-engulfment or phagocytosis remain elusive. One potential candidate mechanism is preservation of Fc -receptor complex at the plasma membrane as suggested by Bergin *et al*., who demonstrated A1AT-mediated TACE inhibition was associated with increased FcγRIIIb expression on neutrophils membrane and decreased neutrophil chemotaxis [14]. Another putative mechanism may be explained by direct functional interaction of A1AT with complement protein, C3b as recently reported in abstract form [37] and may enhance phagocytosis of opsonized bacterial targets via complement receptors pathway.

A novel observation reported in this study is that when AM are simultaneously challenged with both apoptotic and phagocytic targets, each target inhibits the effectiveness of overall AM engulfment. Whereas exposure to CS further inhibits, A1AT treatment improves overall AM engulfment even during this complex engulfment environment. Although our *ex vivo* model cannot be directly extrapolated to chronic airway inflammatory conditions—e.g. COPD, AATD, cystic fibrosis, or severe asthma, the airways of affected patients frequently contain both bacteria (e.g. *Haemophilus influenza*, *Pseudomonas aeruginosa*, or *Staphylococcus aureus*) and a large burden of apoptotic cells [38–41]. Our findings, which require validation in an *in vivo* model, are not surprising, since Medeiros *et al*. have demonstrated that apoptotic cells suppress bacteria phagocytosis in a mouse model of pneumococcal pneumonia [18]. On the other hand, our group and others have published that bacterial released products like alginate and pyocynin dampen the ability of AM to scavenge apoptotic targets [26, 42].

Interestingly, a significantly increased phagocytosis was only noted shortly after A1AT supplementation (4 h), but not after prolonged (16 h) treatment, whereas the reverse was true for enhancement of efferocytosis. This suggests differential, time-dependent effect of A1AT on cell function. We and others have noted similar biphasic effects of A1AT on endothelial cells [28] and macrophages (PBDM) responses to inflammation [43]. In the latter, Subramaniyam *et al*. have demonstrated that A1AT administration enhanced LPS-induced TNF-α, IL-6, and IL-8 release at 4 h from human monocytes and neutrophils, but A1AT delayed effect was not investigated [43]. The mechanisms behind these time-dependent effects require further clarification, as they may determine the timing and frequency of A1AT supplementation for various clinical indications.

Nebulized A1AT has been successfully tested in patients with cystic fibrosis and showed that A1AT treatment decreases sputum anti-elastase activity, airway bacterial or neutrophil load in the CF lung [44]. Based on these reports and our current findings of enhanced AM scavenging function without further incitement of inflammation, administration of A1AT to the airways may be considered for active smokers with acute lung bacterial infections to reduce exacerbation duration, severity, and time to recovery.
